# A Case of Refractory Gastric Antral Vascular Ectasia Treated Successfully With Distal Gastrectomy and Billroth II Reconstruction

**DOI:** 10.7759/cureus.34875

**Published:** 2023-02-11

**Authors:** Hideya Itagaki, Katuhiko Suzuki, Tomoyuki Endo

**Affiliations:** 1 Department of Emergency and Disaster Medicine, Tohoku Medical and Pharmaceutical University Hospital, Miyagi, JPN; 2 Surgery, Honjoudaiichi Hospital, Yurihonjou, JPN

**Keywords:** upper gastrointestinal(ugi) bleeding, distal gastrectomy, argon plasma coagulation, diffuse antral vascular ectasia, gastric antral vascular ectasia

## Abstract

Gastric antral vascular ectasia is a rare cause of upper gastrointestinal bleeding and an important cause of transfusion dependence. Although surgery should be considered when patients with gastric antral vascular ectasia become transfusion-dependent even after endoscopic treatment, surgery for such patients with cirrhosis on dialysis has not been reported. Our patient, a 62-year-old man with a history of cirrhosis and chronic kidney failure, experienced recurrent bloody stool. Upper endoscopic findings indicated a diagnosis of gastric antral vascular ectasia; therefore, we initiated therapy with argon plasma coagulation. Anemia developed, and despite a second argon plasma coagulation treatment, it remained difficult to control. During the six weeks of hospitalization, the patient received more than 40 units of red blood cells. The gastroenterologist determined that further treatment with argon plasma coagulation would increase the risk of gastric perforation; therefore, we performed distal gastrectomy with Billroth II reconstruction. The patient was discharged from the hospital 15 days after surgery and had no signs of anemia for more than one year after discharge. The case of our patient shows that although endoscopic therapy is the usual treatment for gastric antral vascular ectasia, surgery should be considered when anemia is difficult to control.

## Introduction

Gastric antral vascular ectasia (GAVE) was first described by Rider et al. in 1953, but its pathophysiological features remain largely unexplored [1]. This disease is a rare but important cause of upper gastrointestinal bleeding because affected patients can become transfusion dependent [2]. Although endoscopic treatment is considered the “gold standard” for GAVE, surgery is considered a last resort in refractory cases [3]. We report the case of a patient with GAVE who twice underwent argon plasma coagulation (APC), an endoscopic treatment, but in whom GAVE recurred and transfusion-dependence developed; therefore, we performed pyloric gastrectomy with Billroth II reconstruction to control bleeding.

## Case presentation

A 62-year-old man visited our hospital emergency department because of anemia and bloody stool. The patient had been admitted to our gastroenterology department approximately three weeks earlier for anemia (hemoglobin [Hb]: 6.6 g/dL). After admission, upper endoscopy revealed hemorrhagic gastritis, and the patient underwent a blood transfusion and was discharged two weeks later. However, bleeding started immediately after discharge, and a blood test performed at his local doctor's office the day before his visit to the emergency department revealed that his Hb level had dropped to 4.0 g/dL. Therefore, a recurrence of gastrointestinal bleeding was suspected, and the patient was referred to our emergency department the next day.

**Figure 1:**
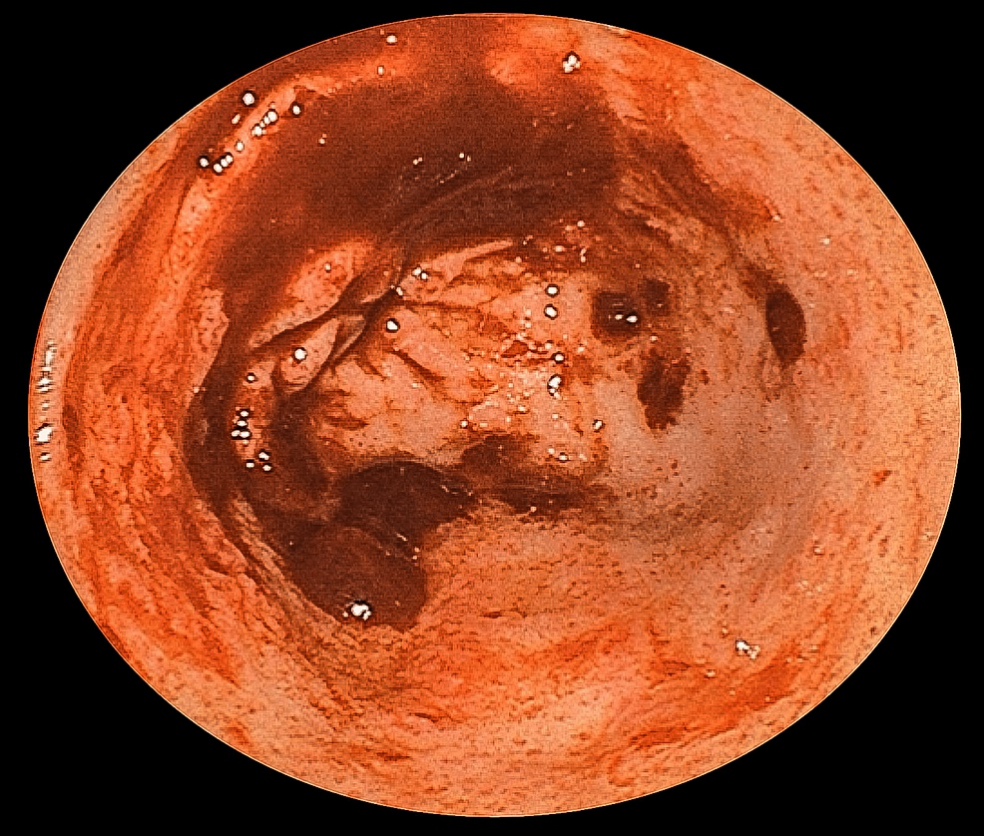
Blood clots circumferentially adherent to the pyloric region of the stomach.

The patient had no related family history, but his medical history included diabetes, chronic kidney failure (treated with hemodialysis), prostate cancer, chronic hepatitis B, and cirrhosis; his Child-Pugh score was B. He was taking rosuvastatin, cinacalcet hydrochloride, voglibose, alogliptin benzoate, glimepiride, enzalutamide, and vonoprazan fumarate (a potassium-competitive acid blocker) [2], all of which had been prescribed at other hospitals. Physical examination on the patient's arrival at our department revealed normal vital signs and no abdominal tenderness; however, the digital rectal examination revealed tarry stool. Blood test results indicated severe anemia (Hb: 4.0 g/L); therefore, we performed an emergency upper endoscopy on the day of his arrival. During this procedure, we observed blood clots circumferentially adherent to the pyloric region of the stomach (Figure 1).

**Figure 2:**
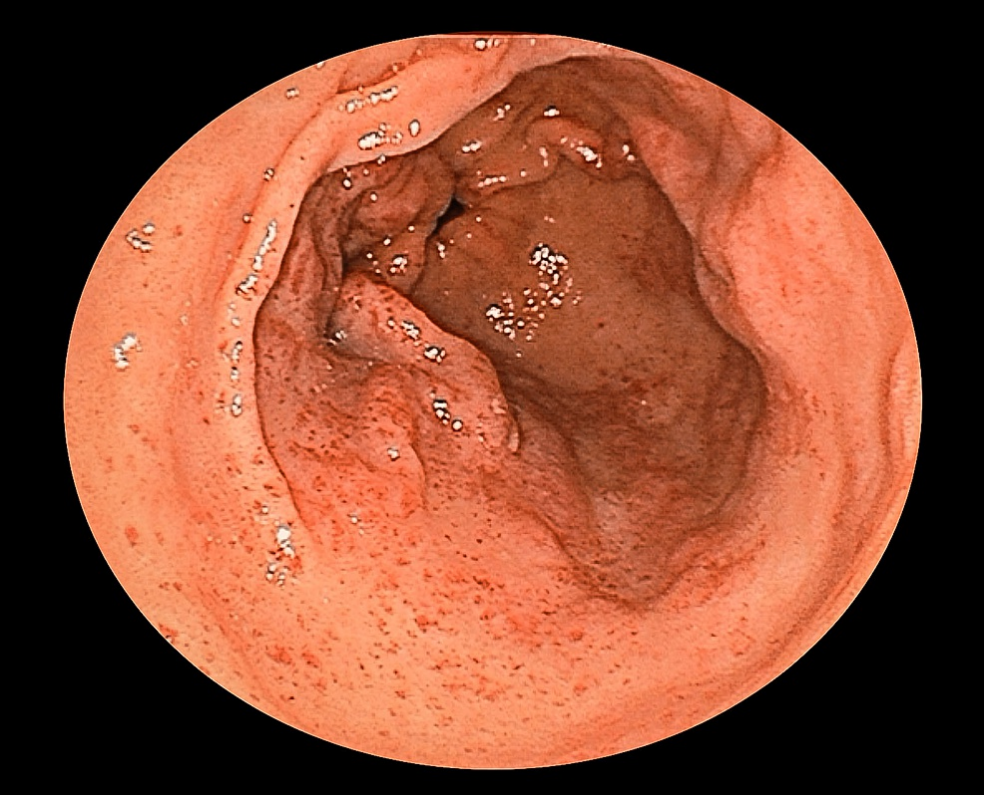
Diffuse redness is visible circumferentially in the pyloric region of the stomach.

Hemostasis was achieved with thrombin therapy, and the patient underwent upper endoscopy again on the third day of hospitalization. Because diffuse redness was present circumferentially in the pyloric region (Figure 2), the patient was diagnosed with diffuse antral vascular ectasia (DAVE), which is considered to be the same disease as GAVE.

**Figure 3:**
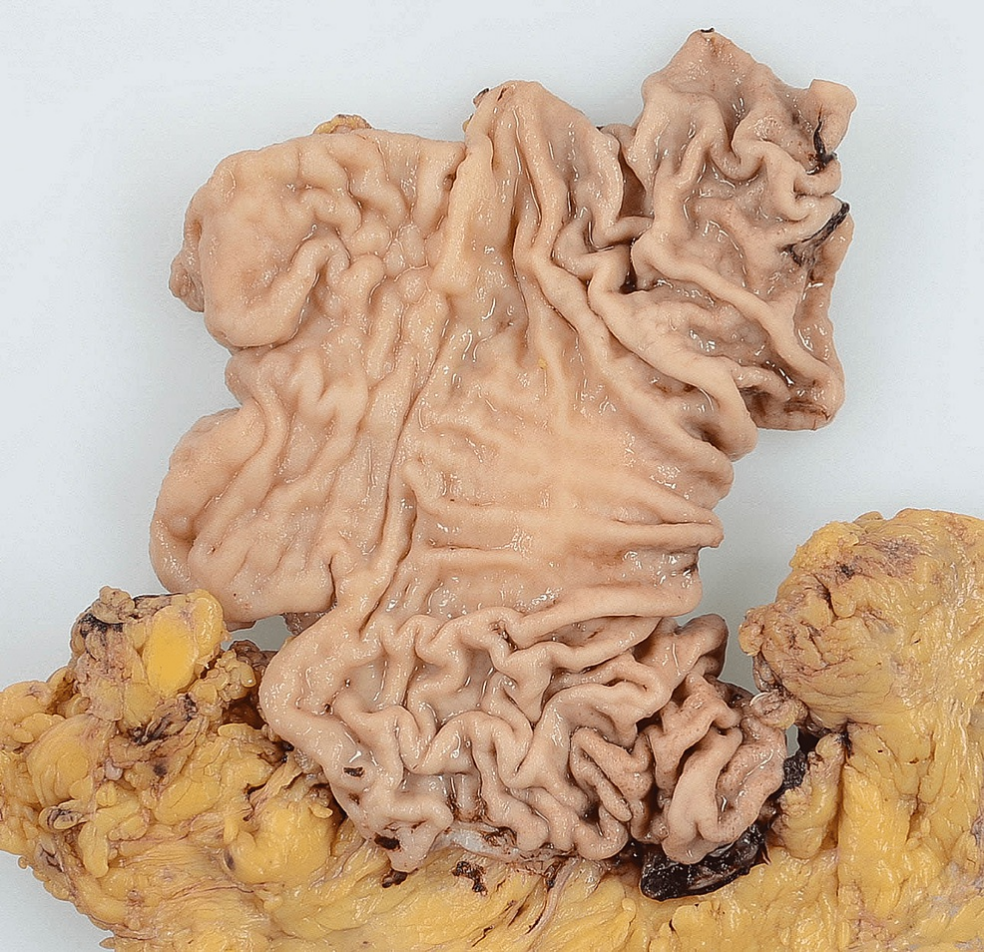
Separated pyloric region of the stomach Findings in the pyloric region resolved after blood vessels were removed to prepare a specimen.

The patient then underwent APC. Bleeding from the mucosa was observed during an upper endoscopic examination on the fifth day of hospitalization, and APC was repeated. Bleeding secondary to DAVE was observed again during upper endoscopy on the 10th day of hospitalization. The gastroenterologist determined that further APC treatment would increase the risk of perforation at many points; therefore, the patient was prescribed an oral proton-pump inhibitor and monitored thereafter. However, he required frequent red blood cell transfusions, and by the sixth week of hospitalization, he had received more than 40 units. Despite the two APC treatments and proton-pump inhibitor therapy, he became transfusion dependent because of refractory anemia. We, therefore, performed distal gastrectomy with Billroth II reconstruction on day 45 of hospitalization (Figure 3). 

**Figure 4:**
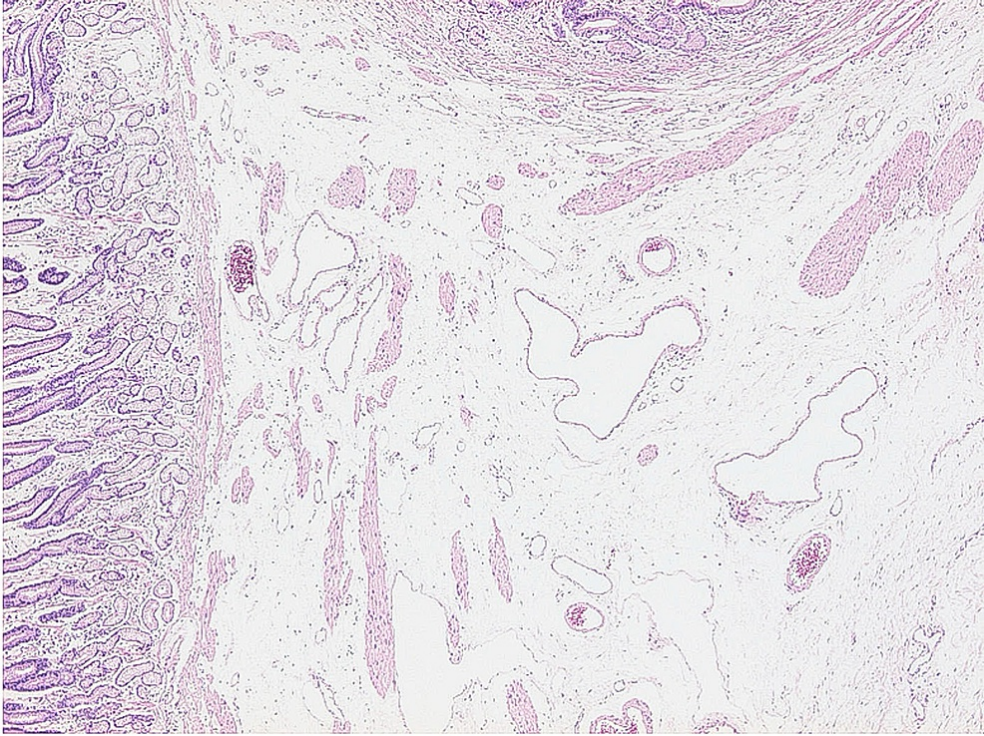
Histopathology of the gastric antrum Findings revealed moderate to severe edema of the mucosa and submucosa, and moderate to severe dilatation of capillaries and veins.

The histological examination revealed moderate to severe edema of the mucosa and submucosa and moderate to severe dilation of capillaries and veins, mainly in the gastric antrum, which led to the pathological diagnosis of GAVE (Figure 4).

On the first postoperative day, the Hb level was 6.9 g/dL. Four units of red blood cells were transfused, and the Hb level rose to 9.0 g/dL. Thereafter, the patient's Hb level remained at 9.1 g/dL, and further no red blood cell transfusion was performed; he was discharged 15 days after surgery.

For more than one year after surgery, no signs of tarry stool or anemia were observed, but one and a half years after surgery, his right femoral neck was fractured in a fall, and he underwent surgery. His liver function deteriorated postoperatively, and he died of liver failure on the 16^th^ day after the surgery.

## Discussion

In our patient, who had cirrhosis and chronic renal failure, GAVE and refractory anemia developed even after two APC treatments, and he underwent surgery. Surgical treatment of patients with cirrhosis is rare, and we have found no reports of surgical treatment of such patients with chronic renal failure who are on dialysis. GAVE, first described as "gastritis characterized by veno-capillary ectasia" by Rider et al. in 1953, is characterized by radial longitudinal vasodilatation of the gastric antrum and is an endoscopic finding known as “watermelon stomach” [2]. Diffuse petechial hemorrhages in the gastric wall, known collectively as “DAVE,” is an endoscopic finding known as a “honeycomb stomach” [4]. However, GAVE and DAVE is considered the same disease because the pathological findings are identical [5]. Among patients with GAVE, the incidence of iron deficiency anemia is 88%, and the incidence of hematochezia is 42%. Oral iron supplements are inadequate for the treatment of anemia, and 60%-70% of affected patients require blood transfusions [2].

The cause of GAVE is still unknown, but the disease is associated with cirrhosis, chronic renal failure, diabetes, autoimmune diseases, hypothyroidism, and cardiac disease [5]. In particular, cirrhosis is found in up to 30% of patients with GAVE, and it is estimated that 2.5% of patients with end-stage liver disease have GAVE [5]. The prevalence of chronic kidney disease in patients with GAVE is not known, but vascular ectasia is a predominant cause of gastrointestinal bleeding in patients with chronic kidney disease, whereas it is not in patients with normal renal function [6]. In our patient, diffuse petechial hemorrhage in the pyloric region led to the diagnosis of DAVE, but cirrhosis of the liver and chronic renal failure may have predisposed the patient to vascular ectasia.

GAVE is typically seen in the gastric antrum and rarely seen in other regions; duodenum, jejunum, and rectum [7,8]. It is necessary to distinguish GAVE from portal hypertensive gastropathy and antral gastritis. Unlike GAVE, the lesions associated with portal hypertensive gastropathy are usually observed endoscopically in the gastric body and fundus. Additionally, GAVE is histologically characterized by capillary dilation and fibrosis of the lamina propria and the presence of fibrin emboli; a differential diagnosis of GAVE is possible based on these observations [9]. 

In our patient, the endoscopic findings were consistent with GAVE because of diffuse petechial hemorrhage in the gastric antrum. Pathological findings also revealed moderate to severe edema of the mucosa and submucosa and moderate to severe dilatation of capillaries and veins, mainly in the gastric antecubital area, and this was pathologically diagnosed as GAVE.

The “gold standard” of treatment for GAVE is endoscopic therapy, mainly thermocoagulation, including APC [10]. Usually, multiple APC treatments are required; according to one study, two or more APC treatments, on average, are necessary to achieve a therapeutic effect in patients with DAVE [11].

Other endoscopic treatments include RFA (radiofrequency ablation) and EBL (Endoscopic Band Ligation). RFA is considered an alternative to APC, but to date, no randomized controlled trials have compared RFA to APC. RFA catheters are used to achieve adequate contact with tissue, and there are through-the-scope catheters with ablation areas of 1.2 cm2, 1.5 cm2, and 2.6 cm^2^, respectively: HALO^60^ and HALO^90 ^[12]. Although the treatment success rate for RFA is reported to be up to 90%, persistent gastrointestinal bleeding can occur even after complete resection with the HALO^90^ ablation system, and 13.3%-40% of GAVE patients remain in need of blood transfusion after RFA [12]. Next, EBL has a treatment success rate of 77.8%-100%, but 15.4%-55.6% of GAVE patients require transfusion after EBL, with a recurrence rate of 8.3%-48.1% [13]. However, in a randomized controlled trial of 88 patients with GAVE assigned to either the APC or EBL group, the EBL group required fewer treatments and fewer blood transfusions [13].

These results suggest that RFA and EBL may be superior to APC in terms of hemostasis, treatment frequency, and recurrence, but the optimal endoscopic treatment for GAVE has not yet been adequately confirmed in high-quality randomized controlled trials, so at present, APC is the first choice for endoscopic treatment of GAVE. APC is currently the first choice for the endoscopic treatment of GAVE.

When anemia is difficult to control with endoscopic treatment, surgery should be considered. Our patient required transfusions with 14 and 26 units of blood before and after the second APC treatment, respectively, and he remained transfusion dependent, which necessitated surgery because the bleeding could not be controlled.

Surgical treatment for GAVE is usually an antrectomy because the lesion is located primarily at the pylorus [14]. However, it is very difficult to determine the appropriate extent of resection during surgery because the pyloric lesion disappears after the vessels have been excised (Figure 3). We performed a Billroth II pyloric gastrectomy to adequately resect the lesion, after which it did not recur. However, surgery in patients with GAVE is highly risky. According to one report of four patients with a history of cirrhosis who underwent pyloric gastrectomy, two died within 30 days of the procedure [15]. The four patients had undergone transjugular intrahepatic pressure shunting, and their portal hypertension did not improve after surgery, which suggests that these patients had extremely advanced cirrhosis. If the portal vein pressure is considerably elevated, clinicians should consider whether surgery is indicated. Prospective studies with larger numbers of patients are needed to determine the ideal protocol for similar patients.

## Conclusions

To our knowledge, surgical treatment in patients with GAVE, chronic renal failure, and cirrhosis who are on dialysis has not been reported previously. Because such patients are prone to bleeding, surgery should be carefully considered when bleeding is difficult to control endoscopically; and patients become transfusion dependent.
